# The Dendritic Cell Dilemma in the Skin: Between Tolerance and Immunity

**DOI:** 10.3389/fimmu.2022.929000

**Published:** 2022-06-28

**Authors:** Nils Scheib, Jessica Tiemann, Christian Becker, Hans Christian Probst, Verena Katharina Raker, Kerstin Steinbrink

**Affiliations:** ^1^ Department of Dermatology, University Hospital, Westfälische Wilhelms-University Münster, Münster, Germany; ^2^ Institute for Immunology, University Medical Center, Johannes Gutenberg University Mainz, Mainz, Germany

**Keywords:** treg cells, tolerance, dendritic cells, cellular immunotherapy, DC targeted vaccines, interleukin 10

## Abstract

Dendritic cells (DC) are uniquely capable of initiating and directing immune responses. The range of their activities grounds in the heterogeneity of DC subsets and their functional plasticity. Numerical and functional DC changes influence the development and progression of disease, and correction of such dysregulations has the potential to treat disease causally. In this review, we discuss the major advances in our understanding of the regulation of DC lineage formation, differentiation, and function in the skin. We describe the alteration of DC in disease as well as possibilities for therapeutic reprogramming with a focus on tolerogenic DC. Because regulatory T cells (Treg) are indispensable partners of DC in the induction and control of tolerance, we pay special attention to the interactions with these cells. Above all, we would like to arouse fascination for this cell type and its therapeutic potential in skin diseases.

## Introduction: DC Subtypes and Ontogeny

In humans, the haematopoiesis is initiated in the yolk-sac around days 16-18 of estimated gestational age (EGA), followed by the migration of immature hematopoietic stem cells, derived from the aorta-gonad-mesonephros, to the definitive location of pre-natal haematopoiesis; the fetal liver (1, 2). The adult myeloid compartment originates from precursor cells within the bone marrow. Common myeloid progenitors (CMP) have the ability to differentiate towards all adult myeloid cells in humans. The myeloid progenitor cell loses its capacity to produce megakaryocytes, erythroid, eosinophil and basophil cells as they become granulocyte macrophage DC progenitors (GMDP). GMDPs eventually acquire the phenotype of macrophage DC progenitors (MDP), which give rise to monocytes or CD123^+^ common DC progenitors (CDP). The latter is capable of differentiating towards CD141^+^ DC type 1 (cDC1) or CD1c^+^ DC type 2 (cDC2), as well as CD123^+^ plasmacytoid dendritic cells (pDC). Villani et al. (3) identified two distinct populations within cDC2 in blood and showed that CLEC9a could serve as a more suitable marker to identify cDC1 compared to CD141. Besides the bone-marrow precursors, monocytes can replenish the DC reservoir especially during inflammation (4). It appears that monocyte-derived DC (moDC) arise primarily from CD14^+^CD16^−^ monocytes and carry the markers CD1c and CD1a in addition to CD11c (**Table 1**).

**Table 1:** Overview of the most important markers of the DC populations.

	moDC	Pre-pDC/AS DC	pDC	cDC1	cDC2	LC
**Transcription factor**	MAFB KLF4	Zbe2 IRF4 KLF4 IRF8 PU.1 Flt3L	IRF8 IRF4 Zeb2 PU.1 Flt3L E2-2	BATF3 IRF8 Zeb2 PU.1 Flt3L Zbtb46 ID2	IRF4 Notch2/KLF4 Zeb2 PU.1 Flt3L Csf-2 Zbtb46 ID2	ID2 RUNX3
**Main phenotypic cell marker**	CD1c+ CD1a+ D11b+ CD103-	CD123+ CD303+ CD304+ CD11c+	CD123+ CD11c+ CD303+ CD304+	CD141+ CD103+ CD11b-	CD1c+ CD5+ CD103+ CD11b+	CD1a+ CD207+ E-cadherin Langerin
**Extended phenotypic cell marker**	CD11c CX3CR1 CD172a (Sirpα) CD64(Fcr1) CD206+	AXL, SIGLEC6 CX3CR1	CD11c CD4+ Btla CD26 Cystatin C CD209 CD17a (Sirpα) Ly6c Clec9a DR6 FCER1 ILT3 ILT7	CD11c Langerin CD24 Btla C-kit Xcr1 CD26 CD36 CD205 Clec9a CADM1 XCR1 BTLA	CD24 Btla C-kit CD26 CD172a (Sirpα) ILT1 DCIR FCER1 CD11c+	CD11c CD11b+ EpCAM CD24 CD205 CX3CR1 CD172a (Sirpα) CD135+ Trop2
**Origin**	BM-HSC	BM-HSC	BM-HSC	BM-HSC	BM-HSC	Embryonic progenitor,may yolk sac
**Differentiate**	Lymphoid and non lymphoid tissue	Lymphoid and non lymphoid tissue	BM	Lymphoid and non lymphoid tissue	Lymphoid and non lymphoid tissue	LN
**Migration/Occurence**	LN, spleen, thymus, blood	LN, spleen,thymus, blood	LN,spleen, thymus	LN, soleen,thymus, blood	LN,spleen, thymus, blood	Through dermis into LN

In humans, cDC1 and cDC2 are located in the dermis. Furthermore, the inflammatory environment in the skin promotes the development of moDC and the recruitment of pDC. The epidermis harbours Langerhans cells (LC) which are marked by the expression of Langerin, CD1a and E-cadherin (5). Although they demonstrate functional characteristics of DC, LC have been reclassified as tissue-resident macrophages (6). The same is true for dermal CD14^+^ cells, previously defined as DC, which most likely represent monocyte-derived macrophages (2). Lineage-tracing experiments in mice deciphered the role of transcription factors in DC development: cDC1 differentiation involves the transcription factors IRF8, IRF4, Id2 (7) and Batf3. Batf3 and IRF8 in particular appear to be indispensable for cDC1 development (5). Differentiation towards cDC2 in mice requires the expression of Id2 and Zeb2 (8), but IRF4 and Notch2/KLF4 expression is also required (5). Differentiation towards pDC requires the expression of IRF8 and IRF4 (5). Furthermore, ZEB2, a protein involved in epithelial-mesenchymal transition, has been described as a critical factor for the development of pDC. Deletion of *zeb2* in murine bone marrow precursors lead to a drastic reduction of pDC *in vitro* and *in vivo*, presumably through the abrogation of *zeb*2-induced repression of *id2*. Loss of ZEB2 favours the development of cDC1 above pDC and appears to be of critical importance for the fate choice of CDP. Finally, moDC formation depends on the transcription factors MAFB and KLF4 (5) (**Table 1**).

Yolk-sac derived macrophages serve as transient progenitors of LC, but monocytes derived from the fetal liver replace them later on (9). In the adult skin, LC are self-sustaining with no contribution of bone marrow-derived cells (10). In mice, CSFR-1 receptor engagement (11) as well as transcription of *runx3* and *id2* are substantial in the maintenance of LC homeostasis.

In addition to phenotypic/functional classifications, DC classify by their migratory capabilities and differentiation stage. Attempts have been made to distinguish resident DC from migrating DC based on markers. For example, resident DC (pDC or cDC subgroups such as CD8^+^ DC or CD8^-^/CD11b^+^ DC) in lymphoid organs (12) or migrating DC from peripheral tissues and non-lymphoid organs (including CD103^+^/Langerin^+^/CD11b^+^) can be distinguished (13). However, however, all DC migrate both in steady state and in inflammation. In the blood, a population of pre-DC forms a subset that is functionally distinct from pDCs and cDCs. AXL and Siglec6 (CD327) expressing cells (AS DC) strongly stimulate T cells in lymphoid tissue. AS DC may also represent a mature cDC2 progenitor and are likely in transition to this subgroup (3).

The description of immature (iDC) and mature (mDC) phenotypes primarily refers to the maturation of DC *in vitro*. So-called iDC express CD11c but little major histocompatibility complex class II (MHC-II) and costimulatory molecules such as CD80, CD83 and CD86, while mDC show an upregulated expression of these costimulatory molecules as well as MHC-II (14). Maturation also occurs *in vivo*, of course, and the markers described *in vitro* are similarly altered.

## Key Questions and Future Directions for the Field

The increasing use of scRNA-seq has led to the description of additional DC subtypes. Transcriptomes, however, incompletely reflect the cell state as gene transcription is stochastic, characterized by transcriptional bursts of varying intensity (15) and half-lives of individual mRNA molecules vary considerably (16). Therefore, there is a need to determine whether the putative heterogeneity of DC, as evidenced by scRNA-seq, also reflects functional differences.

## Dendritic Cell Functions

DC are versatile cells that act as sensors for pathogens and gatekeepers of tolerance at the same time. They link innate signals (pattern recognition and early inflammatory mediators) to adaptive immune responses (T cell priming and Treg induction). DC functions are diverse and highly dependent on circumstances such as cytokines, tissue and cell origin. DC properties, including surface molecules and secreted soluble mediators, can result in tolerogenic as well as stimulatory effects. This means that their function can be beneficial in relation to peripheral tolerance induction or disadvantageous as in transplant rejection (17).

DC decide on the quality and quantity of the immune response simply by their degree of activation (**Figure 2**). As professional antigen-presenting dendritic cells (APCs), they take antigens from their environment, process them and provide co-stimulation that can prime naïve T cells unlike any other MHCII-carrying cell in the body.

**Figure 1:**
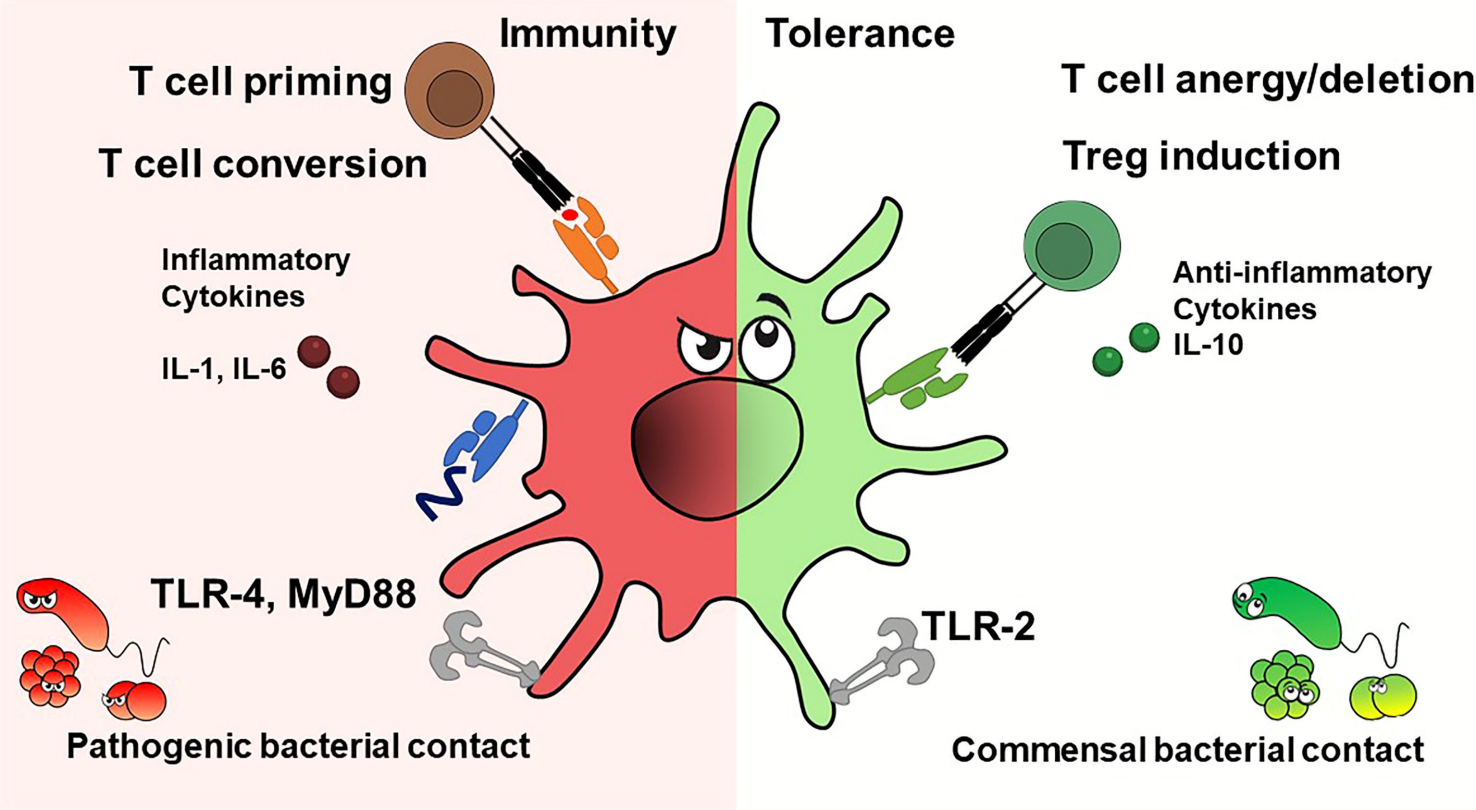
Dendritic cells exhibit immunostimulatory or tolerogenic potential depending on the context. DC's fate depends on cytokines and pathogen contact. Inflammatory cytokines and pathogens presenting TLR ligands promote T effector cells, while anti-inflammatory cytokines or interactions with commensal bacterial components produce tolerance, e.g. induce T cell anergy or deletion, or give rise to regulatory T cells.

Factors that drive DC activation are hallmarks of infection and inflammation such as danger (DAMP) or pathogen associated molecular patterns (PAMP) as well as inflammatory cytokines. DAMPs are molecules or cellular structures released because of cell death or trauma, as heat shock proteins, histones and mitochondrial components. PAMPs are components of pathogens such as LPS, peptidoglycans or bacterial and viral nucleic acids. Recognition of PAMPs or DAMPs *via* toll-like receptors (TLR), C-type lectins or RIG-I-like receptors or sensing of inflammatory cytokines such as tumor necrosis factor (TNF) alpha, interleukins 1 and 6 *via* cytokine receptors (18) induces a differentiation program in DC that has been termed “mature” (18). Such mature DC then express costimulatory molecules, MHC-II and chemokine receptors, which facilitate migration to the lymphoid organs and secretion of elevated levels of inflammatory cytokines. It must be noted, however, that although a DC exhibits characteristics that are termed “mature”, it may have non-immunogenic functions such as tolerance maintenance. At specific anatomical sites (thymus) DC exert tolerogenic functions despite being in a potentially inflammatory environment. Interesting in this regard is the NF-κB pathway. NF-κB is known as a target gene of TNF-α signalling but can also induce TNF- expression during DC maturation. Earlier reports suggest that NF-κB1 is involved in the repression of TNF-α expression in immature DCs by itself (19, 20). Steady-state DC actively induce a tolerance-promoting state (regulatory T-cell (Treg) induction) or render T-cells unresponsive (anergy) (21). Tolerogenic maturation has been shown to depend on IRF4, and ablation of IRF4 in DC impairs peripheral tolerance induction (22, 23) (**Figure 1**).

Interestingly, human tolerogenic DC (IL10-DC) matured *in vitro* in the presence of interleukin-10 induce functional regulatory T-cells regardless of their degree of maturation (CD83 low and high) (24, 25). In addition, DC express distinct toll-like receptors (TLRs), which supports their functional specialization, a process that is highly context- and thus cytokine-dependent. A tolerance-promoting function *via* IL-10 has been reported for (26, 27). Because of its barrier function, the skin also harbours commensal bacteria, which colonize the barrier and thus protect the body from colonization by pathogens. The immune system senses patterns expressed by skin commensals. As previously shown, CD103^+^ DC take up antigens or commensals in the skin or hair follicles and travel to the draining lymph nodes to activate T cells. Primed T cells then migrate back to the skin where they augment the antimicrobial properties of keratinocytes *via* T cell-produced IL-17 (28). Aside from being potent inducers of CD4^+^ and CD8^+^ T cell-mediated adaptive responses, DC can also have direct effects on bacteria. The production of TNF-α and inducible nitric oxide synthase (iNOS) by so-called TipDC has shown that DC can eliminate bacteria in the murine L. monocytogenes infection model. TipDC had allostimulatory capacity in mixed leukocyte reactions but were not required for effective priming of CD4 and CD8 T cells *in vivo* (29). Skin resident LC phagocytes serve as skin guardians, orchestrate immune responses against pathogens, but have also been implicated to induce tolerance to skin’s own antigens under steady-state conditions (30, 31). Local ablation of epidermal LC worsened the dermal phenotype in a lupus mouse model but had no systemic effect on the effects of autoimmune responses. LC regulate skin tolerance *via* IL-10-producing CD4^+^ T cells, but IL-10 levels in skin-draining lymph nodes were unaffected (32) (**Figure 1**).

Human pDC constitutively express the serine protease granzyme B (GrB) (33). GrB production in human pDC is stimulated by IL-21 but inhibited by autocrine production of type I interferons (34). Similarly, IL-10 can increase GrB secretion from pDC, whereas Toll-like receptor agonists and CD40 ligand strongly inhibit them (35). GrB^+^ pDC suppress T cell proliferation in a GrB-dependent, perforin-independent manner, a process reminiscent of Treg. GrB-secreting pDC may therefore play a regulatory role in tumor immune defense, antiviral immune responses, and autoimmune processes. In inflammatory processes, CD8⁺ T cells trigger perforin-mediated apoptosis in pDC, limiting their proinflammatory activity and possibly avoiding autoimmunity (36).

## Key Questions and Future Directions for the Field

Future research in this field must link the phenotypic characterization of the different DC subpopulations to the known functions of DC biology. Is there really a large number of different DC or are there simply states of activation in certain organs at certain times that are functionally indistinguishable?

## Altered DC Function in Disease

As key cells of tolerance and immunity, dendritic cells are directly involved in the development and progression of diseases. Here, we would like to give examples of disease-causing and course-determining DC alterations in cutaneous tumors, autoimmune diseases, and inflammation of the skin.

## Skin Cancer

The DC network of the skin may be one of the first contacts that growing skin tumors have with the immune system. In what way such first contacts influence tumor growth is unclear. However, avoidance of inflammatory responses and immune cell immigration induced by them might help tumors to remain “cold,” i.e., not detected or even ignored by the immune system. If inflammation and immune cell immigration occur, tumors induce numerical and functional changes in progenitor cells and mature DC that attenuate or undermine the immune response against them. Following examples categorize DC alterations in skin cancer:

### Altered DC Recruitment or Survival

Lesions of squamous cell carcinoma (SCC) have significantly reduced numbers of LCs and CD11c^+^ dermal DC, resulting in fewer DC available for effective T-cell priming (37, 38).

Melanomas recruit pDC *via* CXCR4/CXCL12 (39). IFN-induced expression of CXCR3 ligands (CXCL9, CXCL10, and CXCL11) produced by pDC in turn promotes the attraction of additional pDC (40). Through increased attraction, melanomas appear to recruit primarily immature pDC (also called plasmacytoid monocytes) (39).

### Redirection of Monocyte Precursors

IL-6 and macrophage colony-stimulating factor (M-CSF) produced by melanomas promote monocyte differentiation into macrophages rather than DC, limiting effective T-cell responses (41). Melanomas concomitantly produce GM3 and GD3 gangliosides that inhibit DC differentiation from monocyte-derived progenitors and induce apoptosis in monocyte-derived DC (42, 43). Similarly, melanoma-derived cyclooxygenase-1 (COX-1) and COX2 prostanoids block DC differentiation (44).

### Exploitation of Immature DC

Tumor-infiltrating immature DC and pre-DC promote angiogenesis in tumors by secreting endothelial growth factors (e.g., VEGF) and producing other factors that increase the sensitivity of endothelial cells to growth factors (45, 46). Conversely, tumors induce endothelial transdifferentiation in DC progenitor cells, which form a scaffold for the subsequent lining of tumors with endothelial cells (47).

### DC Subversion

Skin tumors undermine anti-tumor immunity not only by altering the differentiation of DC precursors, but also by interfering with the activity of fully differentiated DC:

DC treated with melanoma lysate produce less IL-12p70 albeit without showing changes in maturation markers (e.g., CD40, CD80, CD83) (48). However, melanoma-infiltrating DC also appear to become resistant to some maturation stimuli (49). In turn, DC from regressing melanoma metastases exhibit a stronger T-cell stimulatory potential than DC in progressively growing metastases (50).

More recently melanomas have been found to convert infiltrating cDC2 cells into myeloid cells characterized by decreased CD1c expression and expression of monocyte/TAM markers such as CD14, CD163, CD206, and MerTK (51). Thus, CD14^+^ DC appear to arise not only from monocytes but can also develop from cDC2 in the tumor microenvironment (52). In a three-dimensional melanoma model with multicellular tumor spheroids, lactic acid produced by melanomas suppressed the production of proinflammatory cytokines, including IL-12, by monocyte-derived DC (53).

Melanoma-recruited pDC lose the propensity to produce type I interferons (IFN I), which play an important role in cancer immunization (54, 55). Such altered pDC then support melanoma progression by promoting regulatory immunity through OX40L and ICOSL (56).

Treg contribute to immune tolerance to tumors (57, 58) as evidenced by the fact that their ablation in mouse models improves the anti-tumor immune response (59). However, while early Treg deletion triggers complete rejection of transplanted tumors (60), deletion at later time points only limits tumor growth, suggesting that Treg establish mechanisms in the tumor microenvironment that become Treg independent. Nevertheless, depletion of Treg at late growth phases of tumors also increases the number of functional cytotoxic T cells in tumor tissue and enhances responses to vaccination against tumor antigens (60). However, some observations point to a more complex role for Treg in tumor growth. By limiting inflammatory processes that drive malignant transformation (61), Treg may protect against the development of certain tumor types (62). While sarcomas appear to escape, immune control *via* editing rather than immune repression, lung adenocarcinomas escape because the anti-tumor response itself was suppressed (63). Tumor-infiltrating T cells also often do not appear suppressed, but rather chronically activated and functionally altered (64, 65). Taking into account that Treg primarily form stable contacts with DC in tissues, the effect of Treg in tumors appears to be based less on regulation of T cells and more on DC. How tumor-induced changes in DC affect their interaction with Treg seems for the most part unexplored. It is also conceivable that tumor cells alone do not alter DC in tumors, but interaction with Treg does.

## Autoimmune Skin Diseases

The development of autoimmune diseases bases on alterations in central or peripheral immune tolerance. As early as the 1980s [ten years after their discovery as a separate antigen presenting cell population (66)], DC were observed to act as drivers of T cells in autoimmune lesions (67) and to transfer autoimmunity to naive recipients (68).

Interestingly, observations made in the 1970s (69) that led to the definition of Foxp3^+^ Treg in the 1990s (70) are also closely related to autoimmune diseases. Foxp3^+^ Treg prevent autoimmunity by limiting activation and differentiation of autoreactive T cells through interaction with DC (71), in particular, by regulating the expression of costimulatory molecules (72) and suppressing canonical autophagy (73).

Despite methodological difficulty in reliably identifying human Treg (74, 75), a number of papers have demonstrated that most autoimmune diseases are accompanied by defects in the number or function of peripheral blood Treg, and these observations could be confirmed by *in vivo* disease models (76). Most notably, experimental models demonstrated that attenuation of Treg activity is causal and not a consequence of autoimmune disease (77).

### Preventive Role in Autoimmune Pathogenesis?

Novel models of constitutive and inducible DC ablation and DC-specific gene targeting have further elucidated the role of DC in autoimmune diseases. Enforced antigen expression in steady state DC induces profound and irreversible peripheral unresponsiveness. Both, induction of Treg and anergization or deletion of autoreactive T cells have been shown to be involved in peripheral tolerance induction by DC (21, 78–81). However, constitutive cDC, pDC or CD8^+^ cDC ablation (82–85) results in only a small reduction in Treg numbers but does not cause autoimmunity and DC ablation in an autoimmune-prone background ameliorates rather than exacerbates disease (86). However, these observations do not mean that DC are redundant for peripheral tolerance, because in the absence of DC, effector cells do not form.

Presumably, their functional state rather than their phenotype determines the tolerogenic properties of DC. Although distinct tolerogenic DC can be generated under artificial conditions (87, 88), they are not identifiable in the steady state. It has been suggested that expression of the autoimmune regulator (AIRE), which induces the presentation of a wide range of self-antigens, contributes to central and peripheral tolerogenic DC activity (89, 90), however, in human DC its association with tolerogenic DC activity has been called into question (91).

Independent of a phenotypic distinction of tolerogenic DC populations, there are observations showing that DC counteract autoimmunity by activating regulatory cells. In pemphigus, for example, LC counteract the development of autoimmunity by activating keratinocyte antigen-specific Treg (92).

### Contributing Role in Autoimmune Pathogenesis

While early adoptive transfer experiments had already shown that DC can trigger autoimmune responses (67, 68), a number of anti-inflammatory genes (including *SHP1*, *STAT3*, *αvβ8*-integrin) have since been identified whose absence in DC triggers spontaneous autoimmune responses (93–95). In addition the formation of type I interferons by pDC appears to be involved in all autoimmune processes. Recent observations show that the gut microbiome induces IFN-I production in pDC, which drives a specific epigenomic and metabolic state in cDC that instructs cDC for pathogen control. Interestingly, however, IFN-I-mediated cDC instruction lowers the threshold for self-reactivity (96). In this context, it is noteworthy that of the 81 human autoimmune diseases identified by Hayter and Cook (97), most involve the barrier tissues gut and skin. As in the gut, a continuous confrontation with microbiota takes place in the skin (98). Whether this leads, as in the gut, to an instruction of skin DC is, to our knowledge, not investigated. On the other hand, there are increasing indications that gut commensal-mediated immune instruction becomes systemic and influences immune responses in the skin including autoimmune responses (99).

### Disease Repercussions on DC and Their Significance for Disease Progression

In addition to changes in DC that drive the development of autoimmunity, there are retroactive changes in disease that determine disease progression.

#### DC Accumulation and Altered Cytokine Formation

Accumulation of pDC is characteristic of autoimmune skin lesions. In several autoimmune diseases, increased recruitment of DC to target tissues leads to a decrease in the number of DC in the blood. In rheumatoid arthritis (RA) there is also -presumably in response to the decrease in numbers in the blood- an increased generation of pre-plasmacytoid dendritic cells in bone marrow (100). In systemic lupus erythematosus (SLE) circulating pDCs become functionally abnormal (101).

#### Defective Migration

Local immune activation, in addition to increased attraction of pDC, also ensures that DC already in the tissue increasingly migrate to draining lymph nodes, or, conversely, are retained at the site of inflammation. For example, in autoimmune dermatitis, the migration of LC is impaired (102). The defect in LC migration develops before the onset of skin lesions and correlates with the onset and severity of dermatitis. However, there appear to be both non-migrating LC and migrating cells that look like LC and share some of their characteristics (103).

## Inflammatory Skin Diseases

Inflammatory skin diseases are due to barrier damage or activation of innate and acquired immunity. In contrast to defined autoimmune diseases, inflammatory skin diseases often have an autoimmune component for which it is not conclusively clear whether it is causal.

### Psoriasis

Psoriasis is a common (2% of the U.S. population) chronic inflammatory skin disease characterized by thickening and scaling of the epidermis due to increased proliferation of keratinocytes (104). Psoriasis often develops on damaged skin (Köbner phenomenon), suggesting that innate danger signals may trigger psoriatic inflammation. It is now widely believed that psoriasis is of autoimmune origin (105, 106).

Psoriasis is associated with a greatly increased number of inflammatory DC in diseased skin that act as potent T-cell activators (107). In both psoriasis and relevant mouse models, the IL-23/IL-17/IL-22 axis plays an important role in the disease. In IL-23-induced psoriasis and in Imiquimod-induced psoriasis (a model based on the observation that patients treated with Aldara cream experience flares of psoriasis (108), which most closely match gene expression found in psoriatic skin (109)), the critical DC are TNF-α and IL-1β-producing monocyte-derived DC, including a population of inflammatory Langerhans cells. Flt3L-dependent DC and resident LC appear to be dispensable for inflammation (110).

### Lichen Planus

Lichen planus is a chronic recurrent inflammatory disease of the skin and mucous membranes associated with apoptosis of basal keratinocytes (111). For some time, autoimmune T cells against desmoglein 3- and COL17 have been suspected to play a role in this disease (112). Studies on DC indicate that LC play roles in the pathology of the disease as they accumulate alongside DC in oral lymphoid foci (113). As in virtually all skin autoimmune diseases, in addition to myeloid DC, pDC were observed to secrete IFN-α and granzyme B and possibly help lymphocytes in tissue destruction.

Herpesvirus infections are associated with several autoimmune diseases, including SLE, multiple sclerosis (MS), and RA (114). Importantly, HSV infect DC and alter their function (115). Remissions of lichen planus have been associated with a decrease in protein expression of HHV-7 herpesviruses in pDC (116). Either viruses infect inflamed skin more easily or viral infections lead to the destruction of keratinocytes and thus start reactions against self-antigens.

## Key Questions and Future Directions for the Field

The study of human disease in relation to the role of DC struggles with the chicken-and-egg problem. Are DC drivers of the disease or are changes in DC merely a consequence of the disease? Mouse models of skin diseases such as psoriasis recapitulate the symptoms of psoriatic patients, but are based on triggers that do not occur as such in the pathogenesis of human diseases. In addition, it is unclear which molecules define tolerogenic and immunogenic DC.

## Therapeutic DC Reprogramming

### *In Vivo* Targeting of DC for Therapeutic Approaches

**Figure 2:**
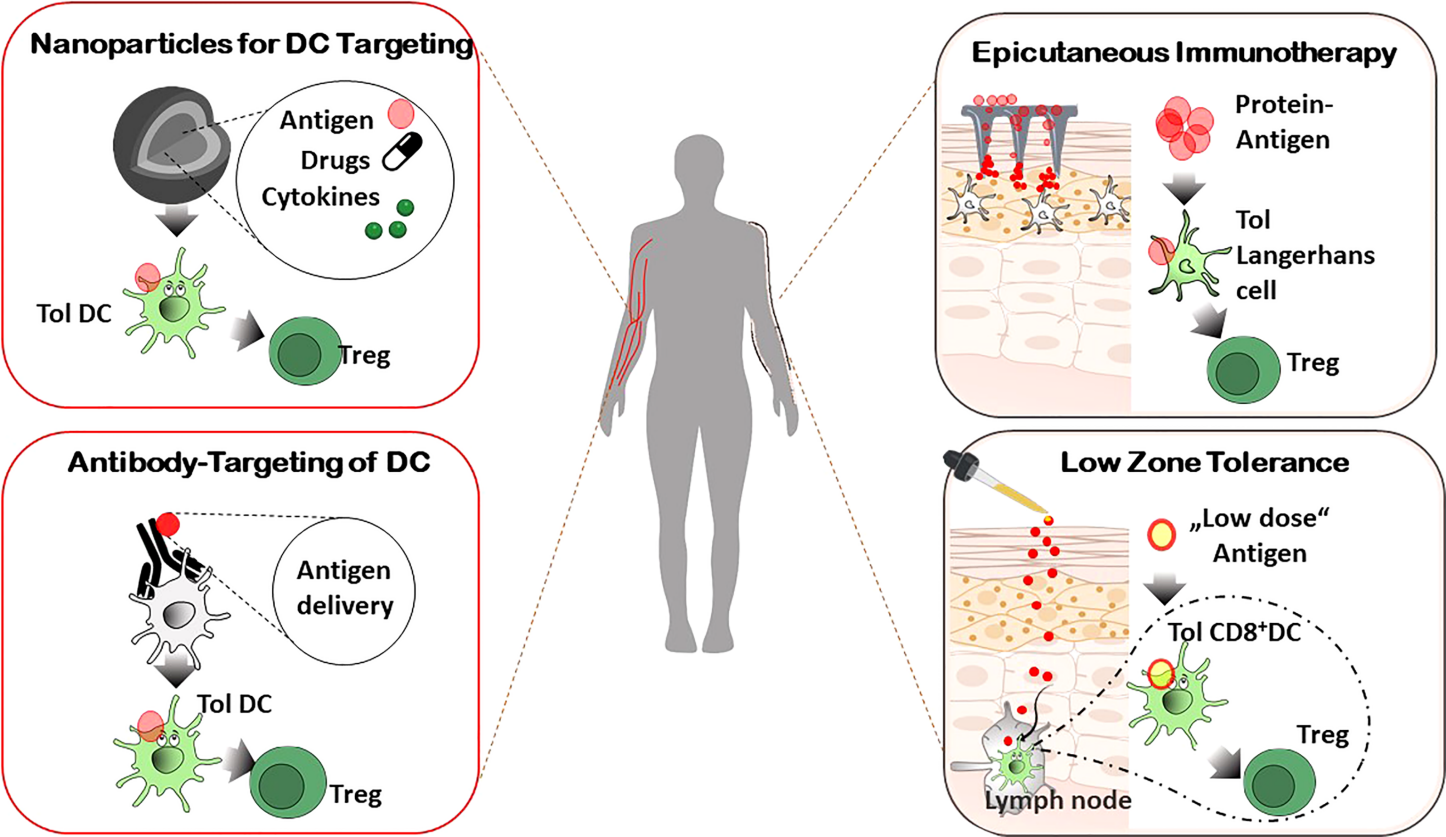
*In vivo* targeting of DC for Treg induction. Systemic administration of specifically designed nanoparticles or antibodies combined with antigen delivery for tolerogenic DC targeting *in vivo*, affects the resulting immune response of allergic and autoimmune diseases in an antigen-specific manner. Epicutaneous application of antigens results in activation of tolerogenic LC in the skin (by dermal patches, Epicutaneous immunotherapy) or tolerogenic DC in skin draining lymph nodes (repetitive exposure to low doses of allergens, Low zone tolerance), respectively, and subsequent control of allergic immune reactions by Treg.

The important potential use of tolerogenic DC in immunotherapeutic applications include allergic and autoimmune disorders and transplantation medicine. Thus, methods for rapid and reliable large-scale production of DC *ex vivo* from peripheral blood of patients have been of great academic and clinical interest and multiple clinical studies have been performed as mentioned below. However, difficulties in obtaining DC from the blood in sufficient numbers, high manufacturing costs and comprehensive requirements for GCP-based production have led to advancements for *in vivo targeting o*f per se tolerogenic DC or for induction of tolerogenic phenotypes *in vivo*, respectively (117–119). In addition to avoidance of costly *ex vivo* isolation, these approaches have the advantage of targeting of DC subsets in their natural environment and offer the availability to a broad range of patients because of donor independency (**Figure 2**).

## Antibody-Mediated DC Targeting *In Vivo*

An alternative strategy for antigen-specific tolerance induction by DC *in vivo* is the use of antigen-delivering antibodies (119, 120). Here, a particular antigen is delivered *via* antibodies that bind to molecules expressed by DC. The rationale is the specific targeting of tolerogenic DC and/or the induction of intracellular tolerogenic pathways after internalization and processing of the antigen (**Figure 2**). So, the processed antigen will be presented in a tolerogenic context, resulting in T cell anergy, T cell deletion, and/or activation and expansion of Treg, respectively. Though many studies focus on the induction of immunity in cancer, *in vivo* targeting of DC was achieved to affect the immune responses in allergies, autoimmune diseases and transplantation rejections as well. Antibody-dependent targeting of DEC-205, an endocytotic receptor highly expressed on cDC1, resulted in antigen-specific unresponsiveness in the absence of adjuvants, whereas presence of maturation stimuli led to pronounced immune responses (21, 118). With regard to the pro-tolerogenic properties of DEC205^+^ DC if given without adjuvants, antibodies against the c-type lectin receptor DEC205 have been administered in different mouse models of autoimmune diseases (e.g. EAE, diabetes, colitis and arthritis) and graft-versus-host disease (121–125), resulting in tolerogenic mechanisms and prevention of inflammatory immune reactions. Dependent on the type of disorder and the antigen used, T cell deletion or T cell anergy of antigen-specific effector T cells and/or Treg activation have been identified as underlying mechanism for tolerance induction. Importantly, a phase I clinical trial in which human DC were targeted *in vivo* with anti-DEC205 linked to the tumor antigen NY-ESO-1 in the presence of maturation factors resulted in a robust cellular and humoral immune response. This proof-of-concept study confirms the preclinical data that *in vivo* targeting of DC leads to an antigen-specific modulation of immune reactions (126). Further studies reported alternative molecules for antibody-mediated targeting of tolerogenic DC as CD207, Treml4, Siglecs, DCIR2, or CLEC9A (DNGR1). Antigen delivery by these antibodies in the vast majority of the studies promoted the activation and/or differentiation of regulatory FOXP3^+^ regulatory T cells (118, 127) (**Figure 2**).

## Use of Nanoparticles for Tolerogenic DC Targeting *In Vivo*

Driven by the initial success in DC-targeting nanocarriers for cancer treatment, promising strategies in this field are under development to modulate the immune system in autoimmunity, allergies, and transplantation medicine (128). DC-specific antibodies can be linked to nanoparticles (NP), allowing for a cell-specific targeting, as well as drug-delivery to induce DC with a tolerogenic phenotype, thereby preventing harmful inflammatory immune responses (**Figure 2**). For successful nanoparticle-based immune modulation through DC targeting *in vivo*, there are some aspects to be considered: 1. physicochemical properties of the NP (material, size, shape, charge, surface modification etc.), 2. disease-specific antigens, 3. DC targeting molecules, 4. co-delivery of drugs or adjuvants with functional properties for tolerance induction and 5. route of administration (119, 128). When using nanoparticles for regulation of immune reactions in autoimmune and allergic diseases, all these parameters can be optimized to boost DC targeting and to control unwanted immune responses. Multiple receptors, including DC-SIGN, mannose and Fc receptors, CD40, or CD11c, respectively, have been used for DC specific targeting in the context of nanomedicine (128). Immunosuppressive drugs and other tolerogenic agents (e.g. siRNA, antisense oligonucleotides) can be packaged into nanoparticles and be co-delivered with (auto-)antigens into DC, resulting in immune tolerance, e.g. by generation and activation of Treg (129) (**Figure 2**). In preclinical *in vivo* models it was demonstrated that poly(lactic-co-glycolic acid) (PLGA) nanoparticles carrying rapamycin were capable of inducing durable immunological tolerance to co-administered proteins *via* induction of tolerogenic DC and subsequent increased numbers of Treg. This immune modulation resulted in an inhibition of antigen-specific hypersensitivity reaction that was superior to the efficacy of rapamycin administered on its own (130). In another approach, nanoparticles were generated that target disease relevant peptides toward MHC class II molecules which then initiate the expansion of antigen-specific regulatory Tr1 cells and regulatory B cells leading to abrogated immunological and clinical symptoms in several murine autoimmune models such as type 1 diabetes, rheumatoid arthritis, multiple sclerosis and inflammatory bowel disease (131). The field of transplantation medicine uses nanoparticles to induce a donor-specific, long-lasting tolerance. Bryant et al. showed that PLGA-based nanoparticles loaded with donor antigens combined with low dose rapamycin at the time of transplant protected transplanted islet allografts from rejection (132).

## The skin as Anatomical Site for DC – Mediated Tolerance Induction

Among specific anatomical sites, the skin represents a crucial barrier that is in constant exchange with exogenous antigens and commensal pathogens, requiring control of immune responses orchestrated by cutaneous and skin-draining DC. As mentioned above, the induction of tolerance vs. immunity depends on the DC subset, immune mediators and receptors involved etc. but as well as on the dose and application route of the antigen. With regard to the antigen dosage, the allergic contact dermatitis (ACD), one of the most frequent occupational skin disorder, is induced in patients and mice by epicutaneous exposure to high doses of contact allergens (haptens). They are taken up by cutaneous DC that prime the allergen-specific ACD effector T cells in the skin-draining LN. A second contact with the same allergen results in activation of these allergen-specifc ACD effector T cells and the clinical manifestations of the ACD (133, 134). In contrast, repetitive epicutaneous applications of very low doses of haptens (low zone tolerance) circumvent the activation of cutaneous DC and in contrast induce an allergen-specific tolerance *via* stimulation of regulatory FOXP3 + T cells and activation and differentiation of CD8^+^ tolerogenic DC in skin-draining LN. The latter ones produce TNF that foster the apoptosis of ACD effector T cells, thereby inhibiting the allergic cutaneous inflammation of ACD (135–137) (**Figure 2**). Thus, the dosage of the contact allergen determines the outcome of the immune response and the development of the resulting skin disease *via* activation of stimulating vs tolerogenic DC, followed by regulatory T cell differentiation respectively.

The idea to modulate an allergic immune response by application of allergens *via* the skin was also further analysed for protein antigens, and called epicutaneous immunotherapy (EPIT) (138). In animal models of food allergies and autoimmune diseases, repetitive applications of an adhesive dermal patch containing antigens protect from inflammation and clinical symptoms *via* uptake of antigens by cutaneous LC and subsequent regulatory T cell activation (119, 138, 139) (**Figure 2**). These promising studies have paved the way for several clinical trials of EPIT that have been completed or are ongoing for milk, peanuts and pollen allergies with encouraging results in terms of safety and tolerability (138).

## Clinical Translation of Tolerogenic DC

For clinical applications, the most available options so far have been to induce immature DC *ex vivo*, and treat them with agents promoting the differentiation into tolerogenic DC phenotypes, which can be given during the entire culture period in the absence of maturation stimuli or only during the maturation phaseThe latter protocols combine the culture of CD14^+^ DC precursor cells in the presence of maturation factors (e.g., LPS or IL-1^+^IL-6^+^TNF^+^PGE2) with additional pharmacological agents, immunosuppressive mediators, exposure to apoptotic cells or gene modification (119, 140, 141). Here, an aberrant DC maturation occurs, resulting in the differentiation of a stable human tolerogenic DC phenotype. Such monocyte-derived DC recapitulate the properties and functionality of naturally occurring DC. Co-culture of monocyte-derived DC with immunosuppressive drugs (e.g. rapamycin, dexamethasone) or cytokines (e.g. TGF-beta, IL-10) or other bioactive agents (e.g. vitamin D3, hepatocyte growth factor, complement factor H, neuropeptide vasoactive intestinal peptide) leads to tolerogenic properties of the differentiated DC populations (140, 142–148). Most of these substances dampen the antigen-presenting properties of DC by reducing expression of costimulatory and MHC class molecules. On the other hand, immunosuppressive surface molecules (e.g. ILTs, PD-L1) or soluble mediators (IL-10, TGF-beta, IDO) are highly expressed by tolerogenic DC subsets (145, 149–151). These differentiated tolerogenic DC are capable to directly suppress the effector functions of other immune cells, such as T or B cells, induce T cell anergy or T cell apoptosis and/or the differentiation and activation of Treg with high suppressive activity.

Since a comparative study by Boks et al. identified IL-10 modulated tolerogenic DC as promising candidates for antigen-specific tolerance induction *in vitro*, we will highlight the generation and properties of these DC subset in more detail. In the first culturing step IL-10 DC differentiate in the presence of IL-4 and GM-CSF to immature DC. The two most prominent protocols differ in the duration of IL-10 supplementation: throughout the culture period (DCIL10) or only during the last two days of culture, in the presence of a maturation cocktail (25, 152–156). Gregori et al. showed that DCIL10 express CD14, CD16 and ILT2-4 but also typical DC markers such as CD83 and CD86 molecules (156). DCIL10 are inducers of regulatory IL10 secreting Tr1 cells that express CD49b and Lag3 and the induction of these Tr1 cells was largely dependent on IL-10 secretion by DCIL10.

In contrast, human IL-10 DC, generated in presence of IL-10 only during the last two days of the maturation phase, were inducers of anergic T cells as well as of Treg, which was independent of IL-10 secretion by DC. The Treg efficiently suppressed syngeneic CD4^+^ effector T cells and cytotoxic CD8^+^ T cells in a cell-to-cell contact dependent and antigen-specific manner (142, 153). The induced T cell anergy was associated with an increased expression of MAP kinase p38 and its effector molecules MEK 2 and 3. The latter ones facilitated the upregulation of the cyclin-dependent inhibitor 1B (p27Kip1), resulting in a cell-cycle arrest in the G1 phase and, thereby in an anergic state of the T cells (154, 155).

As mentioned above, a comparative study by Boks et al. identified IL10-modulated DC (IL-10 DC) as the most potent candidates for tolerance induction as they exhibit important prerequisites for clinical applications in humans such as potent migratory capacity, efficient Treg induction, and the stability of tolerogenic phenotype under inflammatory conditions (146). Investigation of our own laboratory confirmed these theses as we identified two subsets of the tolerogenic IL-10 DC, CD83^high^CCR7^+^ and CD83^low^CCR7^-^ IL-10 DC. Both tolerogenic DC subpopulations exhibited the capacity to induce Treg, but Treg generated in the presence of the CD83^high^ IL-10 DC subset displayed a superior suppressive capacity. In addition, the CD83^high^ DC subset was extremely stable in a pro-inflammatory environment and, due to the increased CCR7, expression showed a very high migratory capacity which is required for DC/T cell-contact in lymphatic tissues (25), identifying this CD83^high^CCR7^+^ IL-10 DC subset as promising candidate for clinical applications.

### Clinical Studies With *Ex Vivo* Generated Human Tolerogenic DC

**Figure 3:**
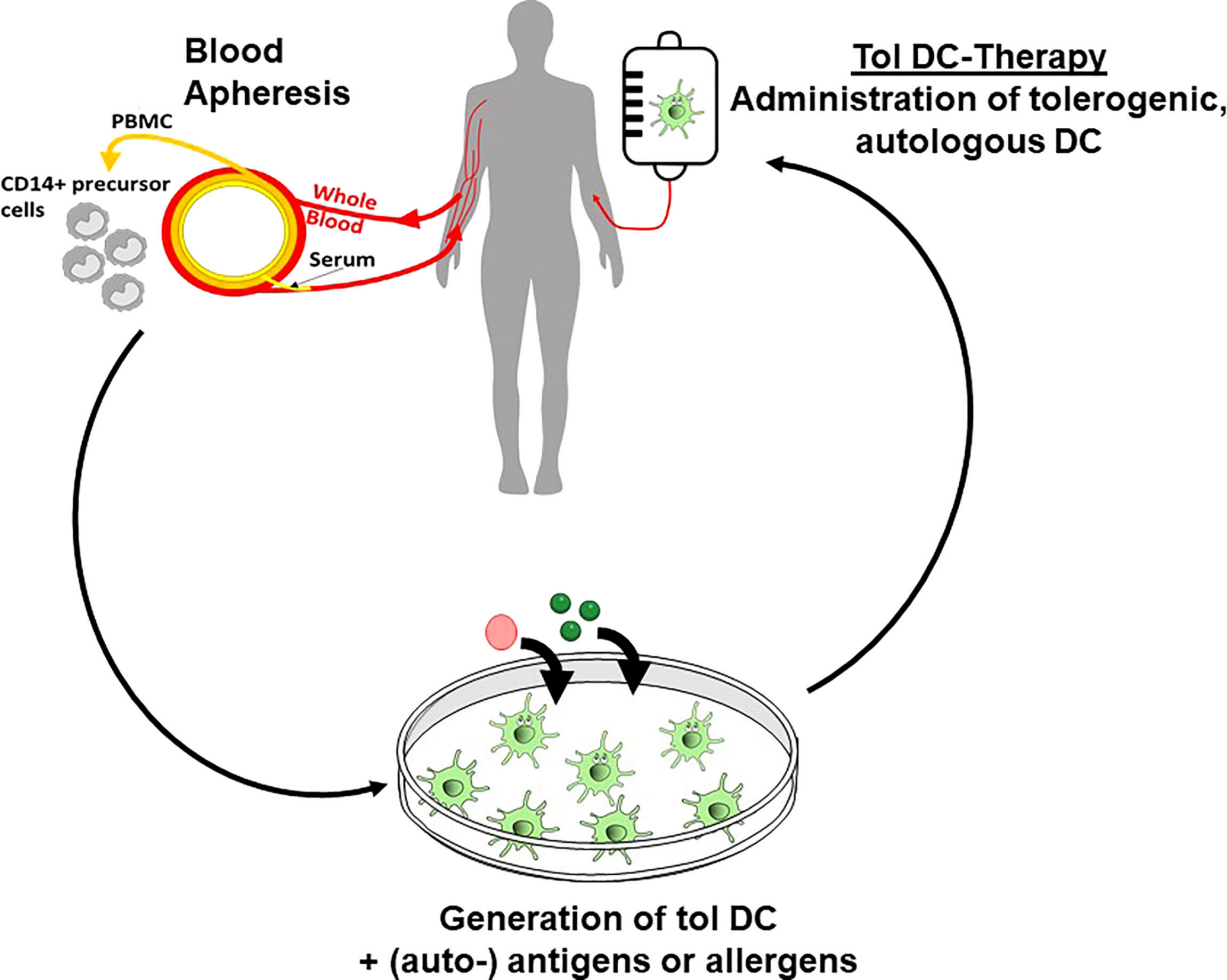
Tolerogenic DC in clinical applications. *Ex vivo* generation of tolerogenic DC starts with isolation of CD14^+^ monocytic precursor cells from the peripheral blood of the patients. After subsequent in vitro culture in the presence of tolerogenic agents and loading with (auto-) antigens, the tolerogenic DC are re-injected into the patients to affect the inflammatory immune reaction in allergic and autoimmune disorders or in transplantation rejection.

The increasing knowledge of tolerogenic DC biology, the promising results from *in vitro* studies with human DC and numerous preclinical animal studies have paved the way for several completed and ongoing clinical phase I trials in autoimmune diseases and transplantation medicine (141) (**Figure 3**). Published studies in patients suffering from diabetes (157), arthritis (158, 159), multiple sclerosis (160, 161) and Crohn´s disease (162) used different protocols for induction of tolerogenic DC phenotypes, including dexamethasone alone or plus vitamin D3 or vitamin A, respectively. Vitamin D3 alone, an NF-kappaB inhibitor, or antisense ODN against CD40, CD80 and CD86 were also used. In the vast majority of the trials the tolerogenic DC were loaded with disease specific antigens/peptides for induction of a specific tolerance reaction. The study results demonstrated a range from no adverse effects until grade 1/2, indicating a high safety profile for tolerogenic DC applications *in vivo*. Analysis of the clinical outcome showed a therapeutic response in a part of the patients, often measured in reduction of disease-specific scores. Immuno-monitoring in some trials revealed a decrease in cytokine production, reduced effector T cell responses, and intriguingly, an increase of the Treg/effector T cell ratio or of the frequency of the Tr1 regulatory T cell subset (158) (**Figure 3**).

In transplantation medicine, data from six phase I/II trails assessing the use of various regulatory cell products, including tolerogenic DC, in kidney transplantation have been analysed. The results demonstrate that regulatory cell therapy is associated with fewer infectious complications, but similar rejection rates, and is therefore a potentially useful therapeutic approach to minimise the burden of general immunosuppression (163). A meta- analysis of 48 records of clinical trials confirmed these data, showing the safety of tolerance-inducing cell-products (including DC) in patients with autoimmune disorders or receiving organ transplantation (160).

## Key Questions and Future Directions

In general, the clinical trials in this field have confirmed the safety and feasibility of autologous *ex vivo* generated tolerogenic DC for therapeutic approaches in autoimmunity and transplantation medicine. However, tolerogenic DC use in real clinical practice has many problems to solve beforehand. Harmonization of study protocols and systematic inclusion of immunological outcome measures would highly improve the development of tolerogenic DC-based treatment approaches. These needs have initiated the implication of consortia in the USA (“The immune tolerance network”) and Europe (“Action to focus and accelerate cell-based tolerance inducing therapies”).

As the generation of autologous *ex vivo* tolerogenic DC exhibit several pitfalls as mentioned, alternative strategies of *in vivo* DC targeting by antibody- and nanoparticle-based techniques or antigen delivery by way of the skin (EPIT, LZT) can be considered for tolerance induction. However, these systems have been primarily tested in preclinical models and further translational trials have to be performed to show the efficacy, feasibility and safety of these novel strategies for tolerance induction *via* DC targeting *in vivo*.

## Author Contributions

CB, VR, and KS contributed to conception and design of the manuscript. NS, CB, VR, and KS wrote the first draft of the manuscript. HP wrote sections of the manuscript. VR designed figures and JT created table 1. All authors contributed to manuscript revision, read, and approved the submitted version.

## Funding

This work was supported by the German Research Foundation (Deutsche Forschungsgemeinschaft, DFG) with TR156/A4/C05-246807620, SFB1009/B11-194468054, SFB1066/B06-213555243 and SFB1450/C06-431460824 (all to KS), TR156/C5-246807620 (to VR), TR156/B02-246807620 (to HP), SFB1066/B08-213555243 (to CB).

## Conflict of Interest

The authors declare that the research was conducted in the absence of any commercial or financial relationships that could be construed as a potential conflict of interest.

## Publisher’s Note

All claims expressed in this article are solely those of the authors and do not necessarily represent those of their affiliated organizations, or those of the publisher, the editors and the reviewers. Any product that may be evaluated in this article, or claim that may be made by its manufacturer, is not guaranteed or endorsed by the publisher.
